# Re: Dao et al., *In situ* observation of urothelial responses to NaCl-induced osmotic stress using optical coherence tomography

**DOI:** 10.1117/1.JBO.31.3.039701

**Published:** 2026-03-01

**Authors:** Pradeep Tyagi

**Affiliations:** University of Pittsburgh, Department of Urology, Pittsburgh, Pennsylvania, United States

## Abstract

The letter comments on a JBO article by Dao et al.

**To the Editor:**

I am writing this letter to shine spotlight on several questionable claims of Dao et al.^1^ The authors used optical coherence tomography (OCT) of excised pig bladder to measure urothelium expansion and shrinkage (Figs. 3 and 4) upon exposure to deionized water and increasing NaCl concentration, respectively, to then conclude: “water transport is…bidirectional” and “presumably triggered by the osmotic gradient” and “osmotic stress of >1.03  Osm/L result in severe damage” (Fig. 5). The reported conclusions are *ex vivo* experimental artifacts of pig bladder excised from perfusion, which are refuted by clinical^2^^–^^4^ and *in vivo*^5^^,^^6^ evidence of well perfused mammalian bladder.

The survival of perfused mammalian bladder^2^^–^^6^ exposed to osmotic stress is dependent upon mucosa (urothelium + lamina propria) receiving threefold to fourfold higher perfusion than detrusor muscle so that circulating blood acts as a dilutional compartment for preventing the local toxic buildup of diffused urine constituents in urothelium.^2^ Perfusion is also essential for transmitting the endocrine action of arginine vasopressin secreted from the hypothalamus, and of aldosterone secreted by the adrenal glands to the bladder. The plasma spikes of arginine vasopressin not only slow the urine production rate of the kidney but also activate aquaporins in urothelium to upload the diffused water from extracellular space of urothelium into the blood by facilitated diffusion.^6^ The diffusion of water from stored urine volume in the bladder manifests as temporary volume reduction (TVR) during sleep, when the rate of urine reabsorption from the bladder exceeds the rate of urine production by the kidney during sleep.^3^ Aldosterone action in the kidney and in the bladder lowers Na+ content of urine, but raises urea content resulting in a hyperosmolar ≤700  mOsm/L morning urine voided by healthy adults after sleep.^6^ As a result, awake human subjects with perfused bladders do not complain of pain upon prolonged exposure to osmotic stress of hypertonic urine up to 1200  mOsm/L and hypertonic 10% to 50% w/v glucose (2.93  Osm/L) during cystoscopic visualization of ureteral jets or hyperosmolar saline infusion during urodynamics.^2^ Meanwhile, the poor bladder perfusion of interstitial cystitis/painful bladder syndrome (IC/PBS) patients plays a pivotal role in their symptoms and in the histological damage comparable to unperfused pig urothelium seen in Fig. 5 of Dao et al.^1^

TVR 20% to 30% each hour measured by periodic bladder ultrasound measurement of healthy human adults during sleep^3^ argues against “bidirectional transport of water”^1^ but favors unidirectional, passive, and paracellular diffusion of water from lumen into the extracellular space of perfused urothelium. TVR^3^ is consistent with first-order kinetics (constant fraction transported each time unit) for water and Na+ transport into perfused urothelium^6^ as opposed to zero-order kinetics (constant amount transported each time unit) of excised pig bladder.^1^ The alleged role of “osmotic gradient”^1^ is refuted by reports of water influx occurring regardless of osmolality following bladder instillation of isotonic saline or isotonic dextrose^5^ or hypotonic fluids and from hypertonic urine during sleep.^3^ Moreover, threefold higher intravesical absorption of Na+ than of a three times bigger dextrose molecule^5^ validates three times faster passive paracellular diffusion of Na+ into urothelium,^5^ which is insensitive to the blockade of epithelial Na+ channel (ENaC) by intravesical amiloride.^5^

Furthermore, mammalian urothelium upregulates ENac expression^5^ to keep the average daily urinary Na+ concentration around 90±38  mOsm/L in IC/BPS patients^4^ and below the isotonic range of 145 mM (145  mOsm/L) in healthy adults and mice.^6^ It is unclear why Dao et al. chose to expose the excised pig bladder^1^ to ≥6 times higher Na+ concentration ≥690  mOsm/L and chose to ignore the role of urea in urine osmolality. Although Dao et al. claimed that their findings carry implications for IC/PBS patients, the claim is questioned by notable deficiencies in their experimental setup and the use of nonphysiological Na+ content.
